# Supplementary material for: Evaluating the screening value of serum light chain ratio, β2 microglobulin, lactic dehydrogenase and immunoglobulin in patients with multiple myeloma using ROC curves
Source: PLoS One. 2023 Feb 16;18(2):e0281743. doi: 10.1371/journal.pone.0281743 (PMC9934331; doi:10.1371/journal.pone.0281743)
Supplement: Attachment — Submitted filename: response to reviewer.docx [file pone.0281743.s002.docx]

Response to Revierwer #1

Dear reviewer,

We really appreciate your review and comments, though we don’t know who you are. We think they’re very constructive. Here’s our point-by-point response.

1. Due to small number of cases, please provide smaple size caluculation to eveluate that the number of patients is optimal for this analysis.

*We used the Medcalc(Version 19.0.4) to calculate the sample size. Details are shown as follows.*

*Basically, we set the type I error as 0.05, type II error as 0.10, Area under ROC curve as 0.875, Null hypothesis value as 0.75, and Ratio of sample sizes in negative/positive groups as 4.*

*The results indicate mumber of positve cases required is 51, and number of negative cases required 204.*

2. Patient in MM group should be exclude patient with SFLC ratio >100 to compare with non-MM group because this study is objective to find factor predictive of MM dignosis. But patient with SFLC >100 can diagnosis of MM and do not need other lab parameters (β2-MG+Ig) to predict the diagnosis.

*We do agree that sFLC ratio >100 is one of the biomarkers indicating active MM according to the IMWG criteria of MM diagnosis. But the sFLC ratio must be interpreted with the condition that clonal bone marrow plasma cells >10% or biopsy-proven bony or extramedullary plasmacytoma. However, the serum light chain assayed in this study is the total light chain instead of the free light chain. So, we suggest patient with serum light chain ratio >100 should be included.*

3. Beta-2 microgloulin might be false high in patients with renal impairment. Therefore please provide baseline creatinine and renal function between both groups.

We provided baseline creatine and renal function between both groups in Table 1. The renal function is calculated by MDRD Study Equatioin.

Best regards,

Linglong Xu
